# Effect of Pre-Season Training on Physiological and Biochemical Indices in Basketball Players—A Systematic Review

**DOI:** 10.3390/sports10060085

**Published:** 2022-05-26

**Authors:** Dimitrios Mexis, Tzortzis Nomikos, Nikolaos Kostopoulos

**Affiliations:** 1 School of Physical Education and Sport Science, National and Kapodistrian University of Athens, 17237 Athens, Greece; dimexis@phed.uoa.gr; 2 School of Health Sciences and Education, Department of Nutrition and Dietetics, Harokopio University, 17676 Athens, Greece; tnomikos@hua.gr

**Keywords:** basketball, pre-season training, physiological, biochemical, assessment

## Abstract

The pre-season period in basketball includes all the physiological attributes that the players need to work on and develop, in order to sustain a full season workload. The monitoring of the effectiveness of pre-season training is based on a variety of biochemical and physiological indices; however, it is still unclear how pre-season training affects those markers. Therefore, this study aimed to analyze the effects of pre-season training on biochemical and physiological markers. A search was performed in five large scientific databases (Pubmed (Medline), Scopus, Science-Direct, Sport-Discus (EBSCO), Semantic Scholar) and produced 7081 results, which after removing duplicates and applying inclusion and exclusion criteria, resulted in 28 published scientific articles being included in this review. The most important findings suggested that the majority of the studies used a 6- or an 8-week pre-season training protocol, because these protocols have shown significant positive effects over the years. In addition, the plyometric training protocols that were used by many studies have been found to be beneficial for basketball athletes for many physiological parameters. Furthermore, the evaluation of biochemical markers can be a very useful tool in monitoring and managing fatigue, which is an essential part of modifying the training process, in order to maximize performance.

## 1. Introduction

Basketball is one of the most popular team sports in the world, and many sport scientists are intensely studying many different aspects and factors that affect or explain players’ performance in this specific sport [1]. Basketball is an intermittent sport, characterized by alternating periods of low and high intensity, and often demanding a range of complex technical abilities, numerous directional changes, and jumps [2]. During basketball practices and matches, both aerobic and anaerobic pathways are heavily triggered, to provide the required energy to the players. Moreover, the capability to maintain a continuous high intensity and to generate strength and power are significant physical determinants of this sport [3], as basketball players have to perform many multidirectional motions at varying speeds and intensities, such as dribbling, shuffling, sprinting, and rebounding [4].

The pre-season period in basketball is considered one of the most crucial phases during a complete basketball season, because it includes all the physical and physiological attributes that the players need to work on and improve, in order to sustain a full season workload. Therefore, in this period, the basic physical skills required in basketball are emphasized during training, along with physiological attributes [5,6]. The pre-season period is often divided into a general preparation phase and a specific preparation phase [7]. The general phase takes place at the beginning of the pre-season and provides a steady increase in training load, in order to avoid any possible injuries. The specific phase takes place right after, and is characterized by heavier training loads and more frequent training units compared to the general preparation phase and the regular season [8]. Although many experts believe that the specific phase is the most important phase of the preparation period, the general phase must also be considered seriously, because the players start training after a long rest period [8].

During the basketball pre-season period, a lot of different types of training and exercises have been used in the past, so that the coaches could maximize the performance of their athletes. Often, these discrepancies can also lead to either under or over-training [9]. Training periodization is a very common way of planning a training protocol, and is widely used in basketball pre-season periods. Periodization is a scheduled workload distribution, focusing on optimizing player performance during the season. Traditional periodization (TP) and block periodization (BP) have been the most common models of periodization training used in basketball over the last few decades [10]. Other known protocols used in basketball pre-season periods to enhance players’ performance include plyometric training and repeated sprint ability training (RSA). Plyometric training is one of the most frequently used methods to develop certain specific abilities, such as strength, power, speed, agility, or vertical jumping, which are essential attributes of many team sports, including basketball [11]. Additionally, the goal of RSA training is to ensure that the players can maintain their maximum performance after numerous high-intensity sprints [12].

The monitoring of the effectiveness of the pre-season training, in terms of performance, on each player, along with the monitoring of the training load and of the fatigue that accompanies this period [13], are important tools for the fitness staff to maximize performance and personalize the training modalities according to each player’s needs [2,14]. This monitoring is based on the determination of a variety of biochemical and physiological markers. However, it is still unclear how pre-season training affects those markers [2]. From this perspective, this study aimed to analyze the effects of pre-season training on biochemical and physiological markers. Basketball coaches will also have the chance to explore the new training regimens and assessment techniques that are being used during basketball pre-season periods. The results of the study may even help sport scientists to create new pre-season training protocols, which may provide additional data for the understanding of the parameters that may enhance or diminish performance in basketball pre-season periods.

## 2. Methods

### 2.1. Protocol Registration

The manuscript is registered in PROSPERO with the registration ID: CRD42022331944.

### 2.2. Information Sources and Search Criteria

An advanced computerized search of the following web databases was performed: Pubmed (Medline), Scopus, Science-Direct, Sport-Discus (EBSCO), and Semantic Scholar. The following combinations of key words were used to perform the searching process: basketball AND (pre-season OR preseason OR pre season), basketball AND (pre-season training OR preseason training OR pre season training). The above terms were searched not only in the articles’ title or abstract, but also within the article. No limitations concerning article type, language, sex, or age were applied in this initial retrieval of articles.

### 2.3. Eligibility Criteria

The eligibility of the retrieved articles was assessed independently by two authors (DM, TN) according to the following criteria: (a) studies written in English, (b) original articles investigating the effect of pre-season training of basketball players on markers of performance, biochemical and psychological status. Studies of both elite and non-elite basketball players were assessed. Exclusion criteria included: (1) age <18 years old, (2) non-English written papers, (3) systematic reviews, trials, books or documents, conference papers, PhD thesis, and reviews. Regarding year of publication, this systematic review had no restrictions, and articles were selected until 7 May 2022. In training terms, many studies were found to include in-season training programs or protocols. These studies were also excluded. Finally, the gender and competitive level of the participants were not considered as exclusion criteria.

### 2.4. Data Extraction and Studies Selection

**Figure 1:**
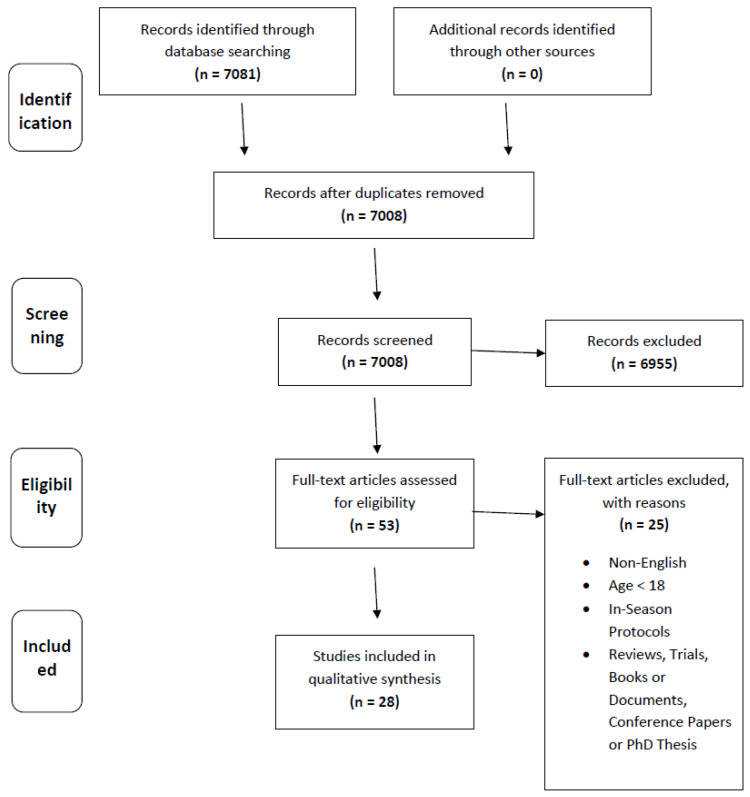
PRISMA Flow Diagram.

A data sheet was created in a spreadsheet file, and all data from the articles were extracted and analyzed. This sheet was created in order to sort all the data, assess carefully if each article met the eligibility criteria and make the required analyses and correlations for the results of the systematic review. Specifically, the following variables were recorded: (1) articles’ analytic information (author’s name, date, journal, volume, issue, pages, doi), (2) volunteers basic characteristics (size, gender, nationality, age, height, weight, competitive level), (3) training protocols (number of protocols, period, type of training, frequency, intensity, duration), (4) physiological/psychological variables (vertical ability, speed, agility, readiness, well-being, etc.), and (5) biochemical variables (metabolic markers, hormones, markers of muscle damage, inflammation, oxidative stress). The 5 databases that were used for this review identified a total of 7081 studies. These studies were extracted to research assistant software Zotero 5.0.94 (Corporation for Digital Scholarship, George Mason University, Virginia, USA) where duplicates were removed. The 7008 remaining studies were then screened for their compatibility, based on our eligibility criteria, which led to the further removal of 6955 studies. The full texts of the remaining 53 studies were downloaded and examined thoroughly; 25 of these studies were rejected because they met our specific exclusion criteria, and the remaining 28 articles were, finally, selected for further analysis (Figure 1).

### 2.5. Assessment of Methodological Quality

Since the majority of the included studies were non-randomized, the methodological index for non-randomized studies (MINORS) was used to assess the quality of the studies (Table 1). In this quality index, twelve methodological items were analyzed, in which a zero score was attributed in cases of no report, 1 point in cases of inadequate report, and 2 points in cases of adequate report. The overall score of each study was assessed independently by two authors (DM, TN). Any disagreement in rating certain items between the two authors was settled through further analysis and discussion.

## 3. Results

### 3.1. Main Characteristics of the Studies

The majority of the studies were published after 2010 (n = 22), which indicates that this specific field has been recently investigated. Despite the increasing popularity in Europe, only nine of the twenty-seven studies were conducted in European Countries (Italy = 3, Poland = 2, Lithuania = 2, Spain = 1, Czech = 1), while seven studies were conducted in the USA. The rest of the studies were reported in various other countries (Brazil = 4, Turkey = 2, Iran = 2, Australia = 1, China = 1, Israel = 1, Tunisia = 1). Regarding the design of the studies, sixteen studies used only one group to assess the differences pre and post the pre-season period, without comparing different training modalities. The remaining studies used 2 or 3 groups (n = 9, n = 3, respectively) to check, not only the differences pre and post the pre-season period, but also to compare the outcomes between different training modalities or between different playing levels (elite vs. non-elite). In addition, the sample size of the studied groups was small (8–19 volunteers), and consisted mostly of the players of the teams that volunteered for each study. As expected, the age of the players was typical for this group of players (18–28). Concerning the gender and the training level of the participants, the majority of the studies included male athletes, while only seven studies included female basketball players, and only one study included mixed athletes [15]. The majority of the studies consisted of elite athletes (n = 18), which is expected, taking into account the importance of the pre-season period in professional basketball (Table 2).

### 3.2. Training Protocols and Duration

Most of the studies used a six (n = 7) or an eight-week (n = 6) pre-season training protocol. These two pre-season periods of training are the most common in elite basketball, so it is logical that most of the studies used these specific periods of time to assess any possible effects on physiological or biochemical indices. Additionally, five studies used a four- (n = 4) or a three-week (n = 1) pre-season training protocol, which in many cases seemed to be inadequate to produce significant results, especially on physiological markers [15,16,17]. On the contrary, three other studies used a twelve-week pre-season training protocol, which is an uncommonly long period. Two of these studies involved professional teams, while the third involved a national team. Furthermore, the majority of the studies used a regular basketball pre-season training protocol (n = 14), which consisted of typical tactical and technical basketball and strength and conditioning training units. On the other hand, seven studies included a plyometric training protocol in their training regimen, accompanied by regular basketball tactical and technical training; as the goal in five of them was to assess the effects of a regular pre-season basketball training program, compared to a plyometric pre-season training protocol accompanied by typical basketball training. The remaining studies tried to evaluate the differences pre and post pre-season period using different training modalities (RSA = 1, rapid strength training = 1, small sided games-SSG and HIT-COD = 1), or to compare a typical pre-season basketball training protocol with some other types of training (RSA = 1, BP = 1, balance training = 1) (Table 3).

### 3.3. Effects of Pre-Season Training on Biochemical and Hematological Indices

In several other studies (n = 6), the researchers collected blood or saliva samples pre and post the pre-season period, in order to evaluate specific biochemical and hematological markers. One study showed a significant decrease in ghrelin (51%) and estradiol (21.5%) levels in female basketball athletes after the pre-season period, but no differences were observed in leptin, adiponectin, and visfatin levels [18]. Moreover, another study presented significant reductions in blood glucose (BG) (−5.8 mg %) and hemoglobin (HGB) (−0.41 mg %) levels after an eight-week regular basketball pre-season period [19]. Additional outcomes for biochemical markers showed that cortisol significantly increased (+75%) after a four-week typical basketball training program in elite male basketball players [16], compared to the study of Andre et al., 2018 [20], that lasted six weeks and demonstrated a significant decrease in cortisol levels (−27.3%) (Table 4).

### 3.4. Effects of Pre-Season Training on Body Composition and Cardiovascular Markers

In total, seven out of 28 studies examined markers of body composition, while one of these studies also examined specific cardiovascular indices. Body composition was assessed mainly through body fat (BF) and body weight (BW) and a variety of methodologies were utilized including skinfolds, densitometry, DEXA (dual-energy radiological absorptiometry), and bioelectrical impedance analysis (BIA). Three studies showed significant decreases in BF (1–4% reduction) after the pre-season period [19,21,22], while two studies demonstrated significant increases in BW (1–1.5 kg), presumably due to increases in lean body mass (LBM) [19,23]. The study by Brown et al., 1974 [19] was the only one that evaluated specific cardiovascular markers, and demonstrated significant decreases in diastolic blood pressure (DBP) (−7.2 mmHg) and recovery heart rate (RHR) (−8.3 bpm) after a typical eight-week basketball pre-season period (Table 3). The results so far are inconclusive for the effect of pre-season training on body composition, taking into account that only few studies assessed this utilizing different methodologies.

### 3.5. Effects of Pre-Season Training on Aerobic and Anaerobic Capacity

Some of the studies (n = 9) assessed the endurance of basketball athletes (aerobic = 8, anaerobic = 3, interval = 1) pre and post the pre-season period. In six out of eight cases where aerobic capacity was evaluated, a significant increase (6–57%) was reported in both elite and non-elite athletes (males and females) under different training modalities. It seems that a pre-season period as short as 3 weeks [23] is able to induce significant alterations in aerobic capacity. Regarding anaerobic capacity, two out of three studies evidenced a significant increase (16–41%) after the pre-season period. The study of Ferioli et al., 2017 [24] was the only one that did not return a significant outcome and consisted of a mixed sample of elite and non-elite basketball players. Based on the same study design, the study of Ferioli et al., 2020 [25] demonstrated a significant increase in anaerobic capacity for both elite and non-elite athletes after the pre-season period, but comparing these alterations the researchers observed that the elite athletes had significantly higher values (+19%) after the pre-season period than the non-elite athletes. Finally, the study of Zeng et al., 2021 [17] used two different training protocols for 4 weeks (SSG and HIT-COD) and assessed the endurance of 19 female basketball athletes through an interval running test. The outcomes of this research demonstrated a significant increase for both groups (4.1 and 4.2%) (Table 3).

### 3.6. Effects of Pre-Season Training on Jumping Ability

Jumping ability is an essential attribute of basketball players, and this was evaluated in eight studies (vertical jump = 8, horizontal jump = 1). The majority of the studies (6 out of 8) demonstrated significant improvements (2–5.50 cm) after the pre-season period. The one study that did not find any significant alterations on vertical ability consisted of both elite and non-elite athletes and lasted eight weeks [25], while a second study consisted of female athletes and lasted 4 weeks [17]. An interesting finding was that, in four studies, different training modalities (plyometric = 2, BP = 1, RSA = 1) were used in comparison with typical pre-season training protocols, and the outcomes showed that in all four cases only the experimental groups (EG) provided significant improvements (2–5.50 cm) in vertical ability versus the control groups (CG) [10,26,27,28]. Finally, the only study that examined horizontal jumping ability [26] showed significant improvements only for the EG (+11 cm), which performed a plyometric training regimen accompanied by the typical tactical and technical basketball training (Table 3).

### 3.7. Effects of Pre-Season Training on Strength and Power

In order to evaluate overall strength, many studies separated the measurements on upper body (UB) strength and lower body (LB) strength [19,21,29]. Additionally, in basketball it is commonly known that the LB part participates more than the UB part. Therefore, when it comes to strength assessment, the majority of the studies examine LB strength or both LB and UB strength. As such, six studies examined LB strength and power, while three of them also examined UB strength. All three studies that assessed UB strength witnessed significant improvements (5.6–11.7 kg), while in the case of LB strength, four out of six studies demonstrated significant increases (5.7–55.2 kg). The two studies that did not find any significant improvements in LB strength and power [15,30] used training protocols with shorter duration, 4 and 6 weeks, respectively, compared with the other four studies that used longer pre-season protocols (8, 8, 8, and 12 weeks). In addition, in the study of Brown et al., 1974 [19], the researchers tried to evaluate grip strength, but the outcomes were not statistically significant. Finally, one study attempted to evaluate specific strength–speed abilities, such as the height of rise of body mass center (Hmax), maximum speed (Vmax), maximum force (Fmax), and maximum power (Pmax), and demonstrated significant improvements after an eight-week specific pre-season training protocol that included 25 plyometric training units [31] (Table 3).

### 3.8. Effects of Pre-Season Training on Speed and Repeated Sprint Ability (RSA)

As was expected, five studies attempted to examine the speed of basketball athletes’ pre and post the pre-season period, mostly through linear sprinting tests. The only study that evidenced significant improvements in speed ability (1.42 s) used a plyometric training program [26]. Additionally, in two other studies, a four-week pre-season training program was used, but the results suggest that a short-term training protocol was not efficient to improve the speed of female basketball athletes [17,32]; compared to the study of Asadi et al., 2017, [26] which used a different training modality and a longer training period of eight weeks. In two further studies [28,33] with the same RSA training protocol, the researchers tried to evaluate RSA, also comparing it with a typical pre-season training protocol [28], and found that the groups that were practicing with repeated sprint training during the pre-season period, showed a significant increase in RSA (0.2 s). Finally, RSA was also evaluated in the study of Zeng et al., 2021, [17] and the results showed that RSA was significantly improved for both groups (2% and 2.1%) (Table 3).

### 3.9. Effects of Pre-Season Training on Balance, Postural Control, and Agility

The study of Lee et al., 2021 [15] used a four-week training protocol, creating three groups (typical training, plyometric training, and balance training), to assess the alterations pre and post the pre-season period in certain physiological attributes, such as balance, and to compare the differences between the groups. No significant alterations were found in any of the groups, and a potential reason might be the short duration of the intervention program. Moreover, postural control was only evaluated by one study [34], which demonstrated significant improvements only by the group that performed the plyometric training sessions (2.7–6%) (Table 3).

Although agility is also a very important skill for basketball athletes, only seven studies out of twenty-eight attempted to evaluate this specific attribute, and only four of them provided significant improvements. The two studies [21,29] that recorded significant improvements (1.6–7%) in agility after the pre-season period used a typical preparation training protocol, while the studies of Asadi et al., 2017 [26] and Zeng et al., 2021 [17] compared a regular pre-season training protocol with a plyometric training program [26], or a SSG with a HIT-COD pre-season training protocol [17]. In the study of Asadi et al., 2017, the researchers noticed significant improvements in agility, not only after the pre-season period in the EG (8.3%), but also between the tested groups; while in the study of Zeng et al., 2021, there were significant improvements for both groups after the pre-season (5.7–7.2%) but not between them. In addition, the study of Borin et al., 2019 [32] demonstrated a significant decrease in agility after a typical pre-season training protocol (−2.3%). Finally, a possible explanation for the three studies that showed a significant decrease or did not return any significant outcomes in agility [15,30,32], might be the short duration of the pre-season period that was used (6, 4, and 4 weeks, respectively), compared to the other three studies that used longer pre-season training protocols (8, 8, and 12 weeks) (Table 3).

### 3.10. Effects of Pre-Season Training on Neuromuscular Performance and Psychological Indices

Neuromuscular performance is considered to be very important for any athlete, but despite the increasing knowledge of its importance, only three studies tried to evaluate neuromuscular performance during the pre-season period in basketball, using various tests and systems such as Biodex and Forceplates. The study of Wilkerson et al., 2004 [35] was the only one that found significant improvements in neuromuscular performance using a plyometric pre-season training program (8.8–11.6%), compared to two other studies [8,36] that used typical pre-season training protocols. Furthermore, four studies used specific questionnaires, in order to evaluate certain psychological aspects of basketball players’ pre and post the pre-season period, such as readiness, quality of sleep, muscle soreness, well-being, and stress. Significant improvements were evidenced in readiness (15–48%) [13,23] and well-being (10%) [23] of basketball athletes; this demonstrates a better psychological profile after the pre-season period. Moreover, another important finding was the strong correlations between the increase in readiness and the increases in power and jumping [13]. This leads to the conclusion that certain psychological aspects can contribute significantly in the physiological development during a basketball pre-season period (Table 3).

### 3.11. Monitoring of Training Load during the Pre-Season Period

Monitoring an athlete’s training load (TL) provides solid evidence for the management of training periodization in many team sports, including basketball. Therefore, we tracked nine studies that attempted to monitor TL during a basketball pre-season period. Two of these studies [8,24] compared the TL of elite basketball players with the TL of non-elite basketball players. The outcomes reported an indisputably heavier training schedule for the elite athletes compared to the non-elite (+113–117%). Additionally, the studies of Ferioli et al., 2018 and Heishman et al., 2018 [8,13] proved that a significant increase in TL during the pre-season period can lead to significant reductions in power and jumping ability (0.7–2%). Finally, the remaining two studies demonstrated a lighter TL towards the end of the pre-season period [23,36], which is logical, considering that the players must stay fresh and restful for the start of the regular season (Table 3).

### 3.12. Effect of Pre-Season Training on Basketball Skills

Just two studies [17,37] attempted to evaluate certain basketball attributes, such as shooting, passing, dribbling, and defensive movement, by performing a specific rapid strength pre-season training protocol for 12 weeks [37] and a SSG or HIT-COD pre-season training protocol for 4 weeks [17]. The first study provided significant development in basketball players’ shooting performance (5.3–6.4%), and the second provided significant outcomes in dribbling and defensive movement for both groups (2.6–5.8%), but shooting was significantly affected only for the SSG group (22.4%) (Table 3).

### 3.13. Study Quality

The quality of the studies was assessed using the MINORS index. The overall quality of the studies was moderate (score 13.07 of 24), but it was noticed that the newest studies (>2018) had better scores (14.38 of 24), meaning that the quality of these studies has improved overtime (Table 1). Meanwhile, it has to be mentioned that we still need more randomized trials in this field, so we can collect better and clearer results, but it is also understood that these trials are very hard to deploy with elite athletes and elite teams.

## 4. Discussion

The majority of basketball teams, especially at elite level, use a 6- or 8-week pre-season training protocol, because primarily these protocols match the timeline of a regular season basketball calendar, and secondarily they have shown significant positive effects over the years [5]. These findings may explain why thirteen studies of this review used a 6- or an 8-week pre-season training protocol. Moreover, we found that, apart from a regular pre-season training program, seven studies used a plyometric pre-season protocol; these studies implemented plyometric protocols because plyometric exercise has been found to be beneficial for basketball athletes on many occasions [38]; such as for improving jumping ability, which is an essential attribute of basketball athletes [11].

Many studies evaluated jumping ability and LB strength (jumping ability = 8, LB strength = 6); an acceptable explanation is that the LB part of basketball athletes is significantly related to all the important basketball-specific moves, such as change of direction, jumping, or other high-intensity movements, and is often measured through jump or strength tests, such as the counter movement jump (CMJ) [39]. From that point of view, these measurements can provide solid results about the overall performance of basketball athletes. In addition, it was mentioned that nine studies tried to measure endurance, but just three of them assessed the anaerobic capacity of basketball players, which is very strange if we consider that anaerobic metabolism is the dominant energy source of basketball players [40,41].

Moreover, six studies examined specific biochemical markers such as creatine phosphokinase (CPK), lactate dehydrogenase (LDH), testosterone, cortisol, and estradiol; these markers are examined in many sports, because they can assess muscle damage and the state of the metabolism of the athlete, which can help monitor, manage, and maximize the recovery and consequently the performance of the athlete [2,42]. Finally, it was noteworthy that two of the studies that measured cortisol found some interesting results. The study of Hoffman et al., 1999 [16] used a 4-week pre-season protocol and demonstrated a significant increase in cortisol levels, while the study of Andre et al., 2018 [20] showed a significant decrease in cortisol levels after a 6-week pre-season protocol. A possible explanation for these findings is that the athlete’s body may need more than 4 weeks to comply with specific training adaptations, so the body is more stressed and tired with just 4 weeks of pre-season training; thus, the cortisol levels are elevated. After 6 weeks, the athlete may have adapted better to the training schedule and consequently demonstrate decreased levels of cortisol [43,44].

Female basketball players appeared to have similar improvements in almost all the measured variables compared to male basketball athletes. Moreover, one study tried to evaluate hormones such as estradiol, ghrelin, visfatin, adiponectin, and leptin in female athletes’ pre and post pre-season period, which is a very rare scientific approach. We can assume that the researchers measured estradiol because it is the major female sex hormone and an estrogen steroid, which is involved in many metabolism procedures such as the regulation of the menstrual cycle or fat distribution [45]. On the contrary, regarding the other four hormones, we can only assume that the scientists were trying to reveal new biomarkers for the assessment of performance and recovery of basketball athletes.

## 5. Conclusions

This is the first review article that has tried to summarize all the studies that took place during a basketball pre-season period and included physiological and biochemical measurements. In conclusion, it has been mentioned that basketball is mainly an anaerobic sport, but only three studies tried to evaluate anaerobic capacity in the pre-season period; therefore, further research may be required, in order to gain more evidence about the anaerobic metabolism of basketball athletes. Regarding the evaluation of biochemical markers, we noticed that just two studies tried to examine biomarkers through saliva samples. The recent discovery of salivary diagnostic procedures for evaluation of performance and recovery offers unique opportunities for valid and easy assessment. In future research, more studies will have the benefit of including saliva measurements in their design. Finally, it has been discovered that, although this review included three studies with a mixed sample of elite and non-elite athletes, there was only one study that was composed of a mixed sample of female and male basketball athletes. Therefore, future studies should try and create pre-season protocols that consist of a mixed gender sample, as this design would be a unique approach for assessing any possible differences between male and female basketball athletes.

## 6. Practical Applications

This systematic review aimed to provide all the up to date data and knowledge on the effect of pre-season training on physiological and biochemical indices in basketball athletes. It also highlights the research gaps related to this precise period in basketball; thus, subsequent studies will have the advantage of being able to focus their research on specific unexplored pathways. As a more practical result, it has been recognized that a 6- or an 8-week pre-season training protocol induced the most significant improvements in terms of physiological and biochemical parameters. Moreover, the studies that included plyometric training in their training regimen showed promising results, especially for improving jumping ability. Furthermore, the evaluation of biochemical markers can be a very useful tool for monitoring and managing fatigue, which is an essential part of modifying the training process, in order to maximize performance. Finally, the basketball coaches of both elite and amateur teams can enlarge their options, connected to their methodologies, and the duration and the training modalities that are used during a basketball pre-season period. This will help sport scientists to design and implement new interventions that may contribute in the understanding of the underlying mechanisms that affect performance in the pre-season period in basketball.

## Figures and Tables

**Table 1:** Methodological index for non-randomized studies (MINORS).

Study	1	2	3	4	5	6	7	8	9	10	11	12	Score
Brown et al. (1974)	2	0	1	2	0	2	1	0	0	0	0	0	8
Tavino et al. (1995)	2	0	2	2	0	2	0	0	0	0	0	0	8
Hoffman et al. (1999)	2	0	2	2	0	1	2	0	0	0	0	0	9
Wilkerson et al. (2004)	2	1	2	2	0	2	0	0	1	2	2	2	16
Boraczyñski and Urniaz (2008)	2	0	2	2	0	2	0	0	0	0	0	0	8
Marzilli (2008)	2	1	2	2	0	2	0	0	0	0	0	0	9
Khlifa et al. (2010)	2	1	2	2	0	2	2	2	2	2	2	2	21
Plinta et al. (2012)	2	1	2	2	0	2	0	1	0	0	0	0	10
Lehnert et al. (2013)	2	0	2	2	0	2	0	0	0	0	0	0	8
Nunes et al. (2014)	2	1	2	2	0	2	1	0	0	0	0	0	10
Scanlan et al. (2014)	2	0	2	2	0	2	0	2	0	0	0	0	10
Schelling et al. (2014)	2	0	2	2	0	2	1	0	0	0	0	0	9
Asadi et al. (2015)	2	1	2	2	0	2	1	0	1	2	2	2	17
Asadi et al. (2017)	2	2	2	2	0	2	0	1	1	2	2	2	18
Ferioli et al. (2017)	2	1	2	2	0	2	0	1	2	2	2	2	18
Andre et al. (2018)	2	1	2	2	0	2	0	0	0	0	0	0	9
Ferioli et al. (2018)	2	1	2	2	0	2	0	0	2	2	2	2	17
Gantois et al. (2018)	2	2	2	2	0	2	1	1	0	0	0	0	12
Heishman et al. (2018)	2	1	2	2	0	2	0	0	0	0	0	0	9
Pliauga et al. (2018)	2	1	2	2	0	2	2	1	1	2	2	2	19
Savas et al. (2018)	2	1	2	2	0	2	1	0	0	0	0	0	10
Borin et al. (2019)	2	1	2	2	0	1	0	1	0	0	0	0	9
Gantois et al. (2019)	2	2	2	2	0	2	1	1	1	2	2	2	19
Ferioli et al. (2020)	2	1	2	2	0	2	0	2	2	2	2	2	19
Heishman et al. (2020)	2	1	2	2	0	2	0	1	0	0	0	0	10
Lukonaitienė et al. (2020)	2	1	2	2	0	1	1	2	2	2	2	2	19
Lee et al. (2021)	2	2	2	2	0	1	1	0	1	2	2	2	17
Zeng et al. (2021)	2	2	2	2	0	1	1	1	1	2	2	2	18

**Table 2:** Main characteristics of the studies.

Study	Nationality	Study Design	Sample (n=)	Mean Age	Gender	Level
Brown et al. (1974)	USA	1 team (Pre-Post training)	18	-	M	Non-Elite
Tavino et al. (1995)	USA	1 team (Pre-Post training)	9	18–22	M	Elite
Hoffman et al. (1999)	Israel	1 team (Pre-Post training)	10	26.4	M	Elite
Wilkerson et al. (2004)	USA	Parallel, 2 teams (regular basketball training vs. plyometric jump training)	19 (11 EG-8 CG)	19	F	Elite
Boraczyñski and Urniaz (2008)	Poland	1 team (Pre-Post training)	14	20.3	M	Elite
Marzilli (2008)	USA	1 team (Pre-Post training)	14	19.3	F	Elite
Khlifa et al. (2010)	Tunisia	Parallel, 3 groups (regular basketball training vs. plyometric training with load vs. plyometric training without load)	27 (9 EG1-9 EG2-9 CG)	23.6	M	Elite
Plinta et al. (2012)	Poland	1 group (Pre-Post training)	16	21.8	F	Elite
Lehnert et al. (2013)	Czech	1 group (Pre-Post training)	12	24.3	M	Elite
Nunes et al. (2014)	Brazil	1 team (Pre-Post training)	19	26	F	Elite
Scanlan et al. (2014)	Australia	1 group (Pre-Post training)	8	26.3	M	Non-Elite
Schelling et al. (2014)	Spain	1 group (Pre-Post training)	8	27.8	M	Elite
Asadi et al. (2015)	Iran	Parallel, 1 team-2 groups (regular basketball training vs. plyometric neuromuscular training)	16 (8 EG-8 CG)	20.3	M	Non-Elite
Asadi et al. (2017)	Iran	Parallel, 1 team-2 groups (regular basketball training vs. plyometric training)	16 (8 EG-8 CG)	18.5	M	Elite
Ferioli et al. (2017)	Italy	Parallel, 2 groups (elite vs. non-elite)	32 (18 non-elite-14 elite)	24.4	M	Mixed
Andre et al. (2018)	USA	1 group (Pre-Post training)	12	-	M	Elite
Ferioli et al. (2018)	Italy	Parallel, 2 groups (elite vs. non-elite)	28 (16 non-elite-12 elite)	24.9	M	Mixed
Gantois et al. (2018)	Brazil	1 team (Pre-Post training)	11	21.5	M	Non-Elite
Heishman et al. (2018)	USA	1 group (Pre-Post training)	10	20.9	M	Elite
Pliauga et al. (2018)	Lithuania	Parallel, 1 team-2 groups (TP training model vs. BP training model)	10 (5 BP group -5 TP group)	21.5	M	Elite
Savas et al. (2018)	Turkey	1 team (Pre-Post training)	13	26.9	M	Elite
Borin et al. (2019)	Brazil	1 team (Pre-Post training)	13	25.3	F	Elite
Gantois et al. (2019)	Brazil	Parallel, 2 groups (regular basketball training vs. repeated sprint training)	17 (9 EG-8 CG)	21.2	M	Non-Elite
Ferioli et al. (2020)	Italy	Parallel, 3 groups (elite I vs. elite II vs. non-elite)	38 (13 elite I-13 non-elite-12 elite II)	25	M	Mixed
Heishman et al. (2020)	USA	1 group (Pre-Post training)	14	19.7	M	Elite
Lukonaitienė et al. (2020)	Lithuania	Parallel, 2 teams (U18 preparation training vs. U20 preparation training)	24 (12 U18-12 U20)	18.8	F	Elite
Lee et al. (2021)	Turkey	Parallel, 3 groups (regular basketball training vs. plyometric training vs. balance training)	25 (9 EG1-8 EG2-8 CG)	18	Mixed (14 F-11 M)	Non-Elite
Zeng et al. (2021)	China	Parallel, 1 team-2 groups (small sided games vs. HIIT-COD)	19 (9 SSG-10 HIT-COD)	19.9	F	Non-Elite

**Table 3:** Studies selected that demonstrate the effects of pre-season training on physiological indices.

Study	Population (Nationality, Sample n =, Mean Age, Gender, Level)	Training Protocol	Duration	Measured Variables (Performed Tests)	Outcomes
Zeng et al. (2021)	China, n = 19, Age = 19.9, Female, Non- Elite	SSG group: typical basketball 4 TU/Week—2 h each TU + 3 SSG TU/Week (2v2 on half court) HIT-COD group: typical basketball 4 TU/Week—2 h each TU + 3 HIT-COD TU/Week (intermittent running with COD)	4 weeks	HR, RPE, 30-15 intermittent fitness test (IFT), RSA, Agility (MAT), 20 m. sprint, Vertical Ability (CMJ), 1 min shooting test, shooting accuracy test, passing test, defensive movement test, control dribble test	SSG: 30-15 intermittent fitness test ↑, RSA ↑, MAT ↑, 1 min shooting ↑, defensive movement ↑, control dribble ↑ HIT-COD: 30-15 IFT ↑, RSA ↑, MAT ↑, defensive movement ↑, control dribble ↑ SSG vs. HIT-COD 1 min shooting ↑
Wilkerson et al. (2004)	USA, n = 19, Age = 19, Female, Elite	CG: stretching, isotonic strengthening, periodic plyometric jumping drills EG: plyometric jump training, stretching, isotonic strengthening	6 weeks	Neuromuscular performance → Hamstrings and quadriceps isokinetic PT (Biodex), Impact force (FSDT and FLT on forceplate system), Agility (TDT)	Post vs. Pre EG: Hamstrings PT ↑ EG vs. CG Hamstrings PT ↑
Marzilli (2008)	USA, n = 14, Age = 19.3, Female, Elite	Normal pre-season program (aerobic running, sprints, basketball drills) + resistance training at maximum intensity	8 weeks	BW, BF% (skinfolds), LBM, Vertical Ability (SVJ, AVJ), Agility (SEMO), UB Strength (1RM Bench Press), LB Strength (1RM Squat)	BF ↓, SVJ ↑, Agility ↑, 1RM Squat ↑, 1RM Bench Press ↑
Nunes et al. (2014)	Brazil, n = 19, Age = 26, Female, Elite	Week 1–3: 3 endurance TU/Week (moderate intensity) + 4 resistance TU/Week (moderate to high intensity) + basketball training Week 4–6: 3 interval endurance TU/Week (moderate to high intensity) + 4 resistance TU/Week (moderate intensity) + basketball training Week 7: 1 interval endurance TU/Week (high intensity) + 2 resistance TU/Week (low intensity) + basketball training Week 8–10: 3 speed-agility TU/Week (maximum intensity) + 3 resistance TU/Week (low intensity) + basketball training Week 11–12: 1 speed-agility TU/Week (maximum intensity) + 2 resistance TU/Week (low intensity) + basketball training	12 weeks	TL (sRPE), Recovery-Stress (RESTQ), UB Strength (1RM Bench Press), LB Strength (8RM Squat), Agility (TDT), Endurance (Yo-Yo), Jumping Power (SJ)	1RM Bench Press ↑, 8RM Squat ↑, Agility ↑, Yo-Yo ↑, SJ ↑
Borin et al. (2019)	Brazil, n = 13, Age = 25.3, Female, Elite	67 total training hours: basketball 73.7%-physical 5.7%-preventive 10.5%-general and special warm-up 10.1%	27 days	TL (PSE), Speed (20 m. sprint), Acyclic Speed (40 m. TDT)	Speed ↓, Acyclic Speed ↓
Lukonaitienė et al. (2020)	Lithuania, n = 24, Age = 18.8, Female, Elite	U18 team: 10 total strength and conditioning TU (57-149′) + 18 total basketball TU (44-131′) + 1 test day (104′) + 5 friendly games U20 team: 7 total strength and conditioning TU (85-128′) + 15 total basketball TU (66-126′) + 1 test day (104′) + 5 friendly games	21 days	BW, BF% (Tanita), TL → Internal TL (TRIMP, sRPE)-External TL (accelerometer), Readiness (rMSSD), Well-being (questionnaire), Sprint (10–20 m. sprint), Jump (CMJ), Fitness (Yo-Yo)	Post vs. Pre U18: BW ↑, Player Load ↓, TRIMP ↓, sRPE ↓, rMSSD ↑, Well-being ↑, Yo-Yo ↑ U20: BW ↑, rMSSD ↑, Well-being ↑, 10 m. sprint ↓, CMJ ↑, Yo-Yo ↑ U20 vs. U18 TL ↓, TRIMP ↓, sRPE ↓, rMSSD ↑
Lee et al. (2021)	Turkey, n = 25, Age = 18, Male-Female, Non-Elite	CG: regular basketball training + 3 strength & conditioning TU/Week—2 h each TU EG1: balance training 2 TU/Week + regular basketball training + 3 strength & conditioning TU/Week—2 h each TU EG2: plyometric training 2 TU/Week + regular basketball training + 3 strength & conditioning TU/Week—2 h each TU	4 weeks	LB Power (SLTH), Balance (BESS), Reactive Agility (Y agility test)	NS
Brown et al. (1974)	USA, n = 18, Male, Non-Elite	3 TU/Week—1:45′ each TU—moderate intensity Endurance, interval, weight lifting → 1 h Basketball drills → 45 min	8 weeks	BW, BF% (skinfolds), WC, VO2max, Resting HR, Maximum HR, SBP, DBP, RHR, PWC, UB Strength (Elbow Extension-Horizontal Flexion), LB Strength, Grip Strength	BW ↑, BF ↓, VO2max ↑, DBP ↓, RHR ↓, PWC ↑, Elbow Extension ↑ (non-dominant), Horizontal Flexion ↑ (dominant and non-dominant), Leg Strength ↑
Scanlan et al. (2014)	Australia, n = 8, Age = 26.3, Male, Non-Elite	44 total TU General Preparatory Phase: Repeated linear running-Repeated linear sprinting Specific Preparatory Phase: Intermittent running drills-Speed and footwork drills-Visual reaction drills-UB power drills-LB power drills-Repeated multidirectional running and sprinting-Multidirectional shuffling drills-Basketball skill-based drills	7 weeks	TL → Internal TL (sRPE, TRIMP, SHRZ)-External TL (accelerometer)	Correlations External Training Load-sRPE External Training Load-TRIMP External Training Load-SHRZ
Asadi et al. (2015)	Iran, n = 16, Age = 20.3, Male, Non-Elite	CG: 2 standard basketball TU/Week—2 h each TU EG: 2 standard basketball TU/Week—2 h each TU + 2 plyometric TU/Week—1 h each TU	6 weeks	Postural Control (SEBT) → Anterior, Anteromedial, Anterolateral, Medial, Lateral, Posterior, Posteromedial, Posterolateral	Post vs. Pre EG: Anterior ↑, Anteromedial ↑, Anterolateral ↑, Medial ↑, Lateral ↑, Posterior ↑, Posteromedial ↑, Posterolateral ↑ EG vs. CG Anterior ↑, Anteromedial ↑, Anterolateral ↑, Medial ↑, Lateral ↑, Posterior ↑
Gantois et al. (2018)	Brazil, n = 11, Age = 21.5, Male, Non-Elite	6 weeks → basketball and repeated sprint ability training	9 weeks (6 weeks of training)	BW, BF% (DEXA), FFM, VO2peak (maximum incremental test), RSA (6 × 30 m. all out sprints)	RSA ↑, VO2peak ↑
Gantois et al. (2019)	Brazil, n = 17, Age = 21.2, Male, Non-Elite	CG: 3 physical and basketball TU/Week—2 h each TU EG: 3 repeated sprint ability training and basketball TU/Week—2 h each TU	6 weeks	VO2max, RSA (6 × 30 m. all out sprints), RVJA, Vertical Ability (CMJ)	Post vs. Pre EG: RSA ↑, CMJ ↑ EG vs. CG RSA ↑, CMJ ↑
Ferioli et al. (2017)	Italy, n = 32, Age = 24.4, Male, Mixed	Elite: 7 basketball TU/Week + 6 physical fitness TU/Week Non-Elite: 5 basketball TU/Week + 3.6 physical fitness TU/Week	7 weeks	Physical Fitness (Yo-Yo Test, Mognoni’s Test, HIIT), TL (sRPE)	Post vs. Pre Elite, Non-Elite: Yo-Yo ↑ Elite vs. Non-Elite sRPE ↑
Ferioli et al. (2018)	Italy, n = 28, Age = 24.9, Male, Mixed	Elite: 7 basketball TU/Week + 5 physical fitness TU/Week Non-Elite: 5 basketball TU/Week + 3.85 physical fitness TU/Week	7 weeks	BW, BF% (skinfolds), Neuromuscular Performance → CMJ (PPO, PF, jump height), COD (PT), TL (sRPE)	Elite vs. Non-Elite sRPE ↑ Correlations ↑sRPE → ↓strength and power
Ferioli et al. (2020)	Italy, n = 38, Age = 25, Male, Mixed	Elite Group I: 6–10 TU/Week—2 strength TU/Week—1 conditioning TU/Week—60–120′ each TU—1-2 games/week Elite Group II: 6–10 TU/Week—2 strength TU/Week—1 conditioning TU/Week—60–120′ each TU—1 game/week Non-Elite Group: 4–7 TU/Week—2 strength TU/Week—1 conditioning TU/Week—60–120′ each TU—1 game/week	8 weeks	BW, BF% (skinfolds), Physical Fitness (Yo-Yo Test, Mognoni’s Test, CMJ, HIIT)	Post vs. Pre All groups: Mognoni ↑, HIIT ↑ Elite group II, Non-Elite group: Yo-Yo ↑ Elite group I vs. Elite group II and Non-Elite HIIT ↓
Tavino et al. (1995)	USA, n = 9, Age = 18–22, Male, Elite	Weight training 3 times/week-Anaerobic training 5 times/week-Aerobic training 5 times/week-Basketball Scrimmages 2–4 times/week	6 weeks	BF% (densitometry), Aerobic Capacity (BPTT), Anaerobic Capacity (APST)	BF ↓, Anaerobic Capacity ↑
Hoffman et al. (1999)	Israel, n = 10, Age = 26.4, Male, Elite	-	4 weeks	Appetite-Quality of sleep-Muscle soreness-Recovery (Questionnaires)	NS
Boraczyñski and Urniaz (2008)	Poland, n = 14, Age = 20.3, Male, Elite	84 TU: Basketball technique and tactics 49 TU, General Endurance 22 TU, Specific Endurance 19 TU, Global Strength 21 TU, Plyometric Training 25 TU	8 weeks	Strength-Speed Abilities → Hmax, Vmax, Tto, Gde, Fmax, PF, Pmax, Pav (CMJ)	Hmax ↑, Vmax ↑, Fmax ↑, PF ↑, Pmax ↑, Pav ↑
Khlifa et al. (2010)	Tunisia, n = 27, Age = 23.6, Male, Elite	CG: Basketball skills training—6 TU/Week-1:30′ each TU EG1: Basketball skills training—6 TU/Week-1:30′ each TU + plyometric training with load—2 and 3 TU/Week during the first 3 and the last 7 weeks, respectively. EG2: Basketball skills training-6 TU/Week-1:30′ each TU + plyometric training without load—2 and 3 TU/Week during the first 3 and the last 7 weeks, respectively	10 weeks	Jumping Ability (SJ, CMJ, 5JT), Muscle Elastic recoil (CMJ-SJ difference)	Post vs. Pre EG1, EG2: CMJ ↑, SJ ↑, 5JT ↑, CMJ-SJ ↑ EG1 and EG2 vs. CG CMJ ↑, SJ ↑, 5JT ↑ EG1 vs. EG2 CMJ ↑, SJ ↑
Lehnert et al. (2013)	Czech, n = 12, Age = 24.3, Male, Elite	10 TU/Week: 16 plyometric TU (two days/week from the 1st to 4th week and four days/week from the 5th to 6th week) + 16 resistance TU + speed exercises and aerobic endurance 16 TU + basketball skill-based training 37 TU + warm-up matches 9 TU	6 weeks	LB explosive strength (CMJ, TSRUJ), Agility (TDT, HOT)	NS
Asadi et al. (2017)	Iran, n = 16, Age = 18.5, Male, Elite	CG: 3 regular basketball TU/Week—2 h each TU EG: 3 regular basketball TU/Week—2 h each TU + 3 plyometric TU/Week—50′ each TU	8 weeks	Vertical Ability (CMJ), Horizontal Jumping Ability (SBJ), Speed (60 m. sprint), Agility (TDT, IAT), LB Strength (1RM Leg Press)	Post vs. Pre EG: CMJ ↑, SBJ ↑, TDT ↑, IAT ↑, 1RM Leg Press ↑, 60 m. sprint ↑ EG vs. CG CMJ ↑, SBJ ↑, TDT ↑, IAT ↑, 1RM Leg Press ↑, 60 m. sprint ↑
Heishman et al. (2018)	USA, n = 10, Age = 20.9, Male, Elite	Morning or Afternoon: 16 strength and conditioning TU/Week—8 total hours Afternoon: basketball training—2 h/week	5 weeks	Readiness (CNS, Overall), TL → Internal TL (TRIMP), External TL (accelerometer), Jump Height (CMJ), Power (CMJ)	Player Load ↑, Power ↓ ↑Readiness → ↑Jump Height ↑Readiness → ↑Power ↑TL → ↓Jump Height and ↑TRIMP
Pliauga et al. (2018)	Lithuania, n = 10, Age = 21.5, Male, Elite	TP group: 2 power TU/Week—2 power endurance TU/Week—2 basketball-specific aerobic endurance TU/Week—1 day rest/Week BP group: week 1–2 (5 aerobic endurance TU/Week—2 days rest/Week), week 3–4 (4 power endurance TU/Week—2 basketball-specific aerobic endurance TU/Week—1 day rest/Week), week 5–6 (5 basketball-specific aerobic endurance TU/Week—2 days rest/Week), week 7–8 (4 power TU/Week—1 basketball-specific aerobic endurance TU/Week—2 days rest/Week)	8 weeks	Vertical Ability (CMJ), Sprint Performance (20 m. sprint)	Post vs. Pre BP group: CMJ ↑ BP vs. TP CMJ ↑
Savas et al. (2018)	Turkey, n = 13, Age = 26.9, Male, Elite	Week 1–4: aerobic conditioning workouts Week 5–12: basketball training + 4–6 rapid strength TU/Week	12 weeks (pre-test applied at the end of 4 weeks and post-test at the end of 12 weeks)	Shooting → 100 shot test (standing free-throw, jump shots, jump shots vs. 1-1 defense), 10 shot test (standing free-throw, jump shots, jump shots vs. 1-1 defense)	100 Shots Test → standing free-throw ↑, jump shots ↑, jump shots vs. 1-1 defense ↑ 10 Shots Test → jump shots ↑, jump shots vs. 1-1 defense ↑
Heishman et al. (2020)	USA, n = 14, Age = 19.7, Male, Elite	2 groups → 7 guards-7 forwards/centers 22 total TU & 7 CMJ measurements	5 weeks	External TL (accelerometer) → Player load-IMA, Neuromuscular Performance (CMJ) → Jump Height-FT:CT-RSI	Player load ↓ IMA (medium intensity) ↓

APST = Anaerobic Power Step Test; AVJ = Approach Vertical Jump; BESS = Balance Error Scoring System; BPTT = Bruce Protocol Treadmill Test; CNS = Central Nervous System; COD = Change of Direction; CT = Contraction Time; FFM = Fat Free Mass; FLT = Forward Lunge test; FSDT = Forward Step Down Test; FT = Flight Time; Gde = Counter-Movement Depth; HIIT = High Intensity Intermittent Test; HOT = Hexagonal Obstacle test; IAT = Illinois Agility Test; MAT = Modified Agility T-Test; NS = Non-Significant; Pav = Average Power; PF = Peak Force; PPO = Peak Power Output; PT = Peak Torque; PWC = Physical Working Capacity; RESTQ = Recovery-Stress Questionnaire for Athletes; RM = Repetition Maximum; rMSSD = Root of the Mean Sum of the Squared Differences; RSI = Relative Strength Index; RVJA = Repeated Vertical Jump Ability; SBJ = Standing Broad Jump; SBP = Systolic Blood Pressure; SEBT = Star Excursion Balance Test; SHRZ = Summated-Heart-Rate-Zones; SJ = Squat Jump; SLTH = Single Leg Triple Hops; sRPE = Session Ratings of Perceived Exertion; SVJ = Standing Vertical Jump; TDT = T Drill Test; TRIMP = Training Impulse; TSRUJ = Two Step Run Up Jump; Tto = Take-Off Time; TU = Training Units; U18 = Under 18 years old; U20 = Under 20 years old; WC = Waist Circumference; 5JT = 5 Jump Test.

**Table 4:** Studies selected that demonstrate the effects of pre-season training on biochemical indices.

Study	Population (Nationality, Sample n =, Mean Age, Gender, Level)	Training Protocol	Duration	Measured Variables	Outcomes
Plinta et al. (2012)	Poland, n = 16, Age = 21.8, Female, Elite	5 TU and 1 match/Week—2 h each TU—moderate intensity 20 min. warm up—90 min. aerobic running—10 min. jogging	3 months	Estradiol, Leptin, Adiponectin, Ghrelin, Visfatin	Estradiol ↓, Ghrelin ↓
Nunes et al. (2014)	Brazil, n = 19, Age = 26, Female, Elite	Week 1–3 → 3 endurance TU/Week (moderate intensity) + 4 resistance TU/Week (moderate to high intensity) + basketball training Week 4–6 → 3 interval endurance TU/Week (moderate to high intensity) + 4 resistance TU/Week (moderate intensity) + basketball training Week 7 → 1 interval endurance TU/Week (high intensity) + 2 resistance TU/Week (low intensity) + basketball training Week 8–10 → 3 speed-agility TU/Week (maximum intensity) + 3 resistance TU/Week (low intensity) + basketball training Week 11–12 → 1 speed-agility TU/Week (maximum intensity) + 2 resistance TU/Week (low intensity) + basketball training	12 weeks	Testosterone, Cortisol, IgA	NS
Brown et al. (1974)	USA, n = 18, Male, Non-Elite	3 TU/Week—1:45′ each TU—moderate intensity Endurance, interval, weight lifting → 1 h Basketball drills → 45 min	8 weeks	Cholesterol, Blood Glucose (BG), Protein, HGB, HCT, LDH (B-B)	BG ↓, HGB ↓
Hoffman et al. (1999)	Israel, n = 10, Age = 26.4, Male, Elite	-	4 weeks	Testosterone, Cortisol, CPK, Urea, LH, TSH, T3, FT4	Cortisol ↑
Schelling et al. (2014)	Spain, n = 8, Age = 27.8, Male, Elite	13.2 TU/Week → 6.5 physical TU/Week—5.7 basketball TU/Week—1 Game/Week	6 weeks	Testosterone, Cortisol, T-C ratio	NS
Andre et al. (2018)	USA, n = 12, Male, Elite	-	6 weeks	Testosterone, Cortisol, T-C ratio	Cortisol ↓

FT4 = Free thyroxine; HCT = Hematocrit; IgA = Immunoglobulin A; LH = Luteinizing hormone; T3 = Triiodothyronine; TSH = Thyroid stimulating hormone.
